# The Book World of Medicine and Science

**Published:** 1903-01-03

**Authors:** 


### THE BOOK WORLD OF MEDICINE AND SCIENCE.

YEAR-BOOK OF THE SCIENTIFIC AND LEARNED SOCIETIES OF GREAT BRITAIN AND IRELAND. Compiled from Official Sources. Nineteenth Annual Issue. (London: Charles Griffin and Co. 1902. Price 7s. 6d.)

THOSE interested in the progress of science will find this year-book very useful, containing as it does not only a list of the various scientific societies with their addresses, their objects, their places of meeting, and names of their principal officers, but in regard to many of them the titles of the papers recently read before them.

WHO'S WHO, 1903. An Annual Biographical Dictionary. Fifty-fifth year of issue. (London: Adam and Charles Black. 1903. Price 5s. net.)

YEAR by year the world grows more inquisitive, and so, as is shown by the growth of this most useful annual, do the means of satisfying its curiosity. This book is in fact a most wonderful compendium of information, giving personal details concerning such a crowd of people that one cannot but wonder how they were all selected. There certainly is no book like it, and probably there are few, if any, annuals which are more frequently referred to.

AGE AND OLD AGE. A Handbook and Guide to the Care of Old Age in Health and Disease. By DAVID WALSH M.D. (London: R. E. Everett and Co. 1902. Price 2s. 6d.)

THIS is a brightly-written readable book, and is apparently addressed to those who wish to attain old age and to enjoy it, and to those who have charge of old people rather than to medical practitioners. Its pages, however, contain a good deal that may be found of interest to the latter, especially to the younger members of the craft, for sometimes even doctors, in the heyday of health and strength, fail to make full allowance for the lessened activity of the bodily functions which is to be found in those who are growing old. More than half the book is occupied by a somewhat discursive discussion of the various influences which make for premature senility, such as the effects of dissipation, drink, self-indulgence, excessive muscular effort, and of various occupations, in shortening life, and of the things which tend or are imagined to tend to long life. First among the latter one clearly ought to place a judicious selection of one's ancestry! Unfortunately, the matter of heredity is one over which we have no control, but there are many other matters as to which man during his youth and maturity can do much to delay the onset of age, and to ensure that old age when it arrives shall be passed in comfort; and with these matters Dr. Walsh deals with great perspicuity, quoting largely from the late Sir George Humphrey's well-known book on the subject. The last few chapters of the book deal with the maladies of old age, with nursing, diet, clothing, etc., as to all of which Dr. Walsh has much to say that bears the impress of common sense. The best part of the book, as it seems to us, is that which is devoted to the diet of the aged. Amongst his various suggestions therein contained we find a much wider range of substances recommended than is sometimes provided for old people. Dr. Walsh studies toothsomeness as well as mere digestibility, and we have no doubt that when proper care is taken in the preparation of the food he is quite right. For example, he very properly praises Irish stew as an excellent dish for old folk. His great forte, however, is "sandwiches." He says that "no one has yet gauged the possibilities of the sandwich in feeding the sick and aged." The bread must be stale enough to cut into thin slices without crumbling; these must be cut free from crust, and one or both of them may be thinly buttered according to taste. Cold roast beef, corned beef, veal, mutton, chicken or game may be served in this way, being either cut in thin shavings entirely free from gristle, or finely minced or pounded before being spread upon the bread. Salt should be added and black pepper in coarse grains from a pepper mill is a useful condiment, serving as it does to prevent or relieve flatulence. Liver makes a good sandwich, and perhaps one of the best ways of giving it is as *pâté de foie gras*, "which is not only very nutritious but also as a rule readily digestible." A layer of Bovril placed between two layers of white or brown bread, thinly spread with butter or cream, is a form of condensed food, we are told, which old people can take with enjoyment. Then most forms of cold fish can be used in the same way, with a little "real melted butter" and salt and pepper. Oysters lightly stewed in milk, or a small slice of lobster, hardly thicker than a shilling, or sliced prawns, can be taken with relish by old persons in the form of a sandwich, as also may many kinds of fruit with sugar and cream or butter. One can evidently, by following out Dr. Walsh's suggestions, devise an endless variety of tasty little "snacks" that old people will often take when "meals" would be positively refused. Dr. Walsh's remarks about clothing are also good. Certainly to those who wish to become old comfortably, this little book presents many points of interest.

ADVANCED HYGIENE FOR THE ADVANCED EXAMINATION OF THE BOARD OF EDUCATION. By ALFRED E. IKIN, B.Sc. (Lond.) L.C.P., Principal of Kettering Pupil Teachers' Centre, and ROBERT A. LYSTER, M.B., B.Ch., B.Sc., D.P.H., Principal of the Municipal Technical School, Smethwick, pp. 300, and 87 figures. (London: University Tutorial Press, Limited. 1902. Price 3s. 6d.)

THIS little handbook is manifestly designed to meet the necessities of the examination room. It is intended to serve as a guide to students preparing for the advanced examination of the Board of Education. Most of the books already available for this purpose the authors consider cover far more ground than the average student finds it possible to assimilate in one session. This perhaps may be so, but we venture to think that that should hardly afford argument for an advocacy of "the irreducible minimum" of information. There is no doubt this little work tends to encourage study for the sake of examinations rather than the value of knowledge. But so far as this so-called "advanced hygiene" goes it is not without merit for it presents many useful facts in a concise and fairly accurate manner. The directions for practical work at the end of each chapter undoubtedly afford the most instructive portions. The illustrations are all too few, and several of those illustrating the chapter on preventable diseases are very feeble. The work, however, is likely to prove of service to many for whom it was written.

